# Wandering Spleen in an Adult Man Associated With the Horseshoe Kidney

**DOI:** 10.5812/atr.9332

**Published:** 2013-12-01

**Authors:** Mohammadreza Memari, Mohsen Nikzad, Hossein Nikzad, Aliakbar Taherian

**Affiliations:** 1 Najafabad Azad University, Najafabad, IR Iran; 2 Faculty of Medicine, Kashan University of Medical Sciences, Kashan, IR Iran; 3 Anatomical Sciences Research Center, Kashan University of Medical Sciences, Kashan, IR Iran

**Keywords:** Adult, Horseshoe Kidney, Splenectomy, Wandering Spleen

## Abstract

**Introduction:**

A wandering spleen occurs when there is a laxity of the ligaments that fix the spleen in its normal anatomical position.

**Case Presentation:**

This is a case report of a wandering spleen with horseshoe kidney in a 29-year-old male admitted with acute lower abdominal pain and vomiting to emergency department of Shariati hospital in Isfahan province. Sonographic examination showed a homogeneous 21 × 15 × 8 cm mass in the lower part of the abdomen and pelvis associated with a horseshoe kidney. Laparotomy confirmed the clinical and ultrasound findings.

**Conclusions:**

The association of horseshoe kidney with a wandering spleen in this case may be due to an embryological anomaly.

## 1. Introduction

Wandering spleen is displacement of the spleen from its normal location due to a loss or weakening of ligaments that hold the spleen in the left upper quadrant (1). Van Horne described the first case report of wandering spleen in 1667, while performing an autopsy (2). The real incidence of wandering spleen is unknown, as up to 50% of the cases remain asymptomatic. Wandering spleen is a rare entity, with a reported incidence less than 0.2% and accounts for only 2 per 1000 splenectomies and has a female predominance (3-5). Due to the long and mobile nature of the vascular pedicle, the possibility for torsion of the spleen is high. Generally, causes are asymptomatic. In delayed diagnosis situations, as a result of development of vascular congestion associated with chronic torsion, symptoms of splenomegaly, left portal hypertension, gastric fundal varices, pancreatitis and hypersplenism may emerge (6, 7).

## 2. Case Presentation

**Figure 1.**
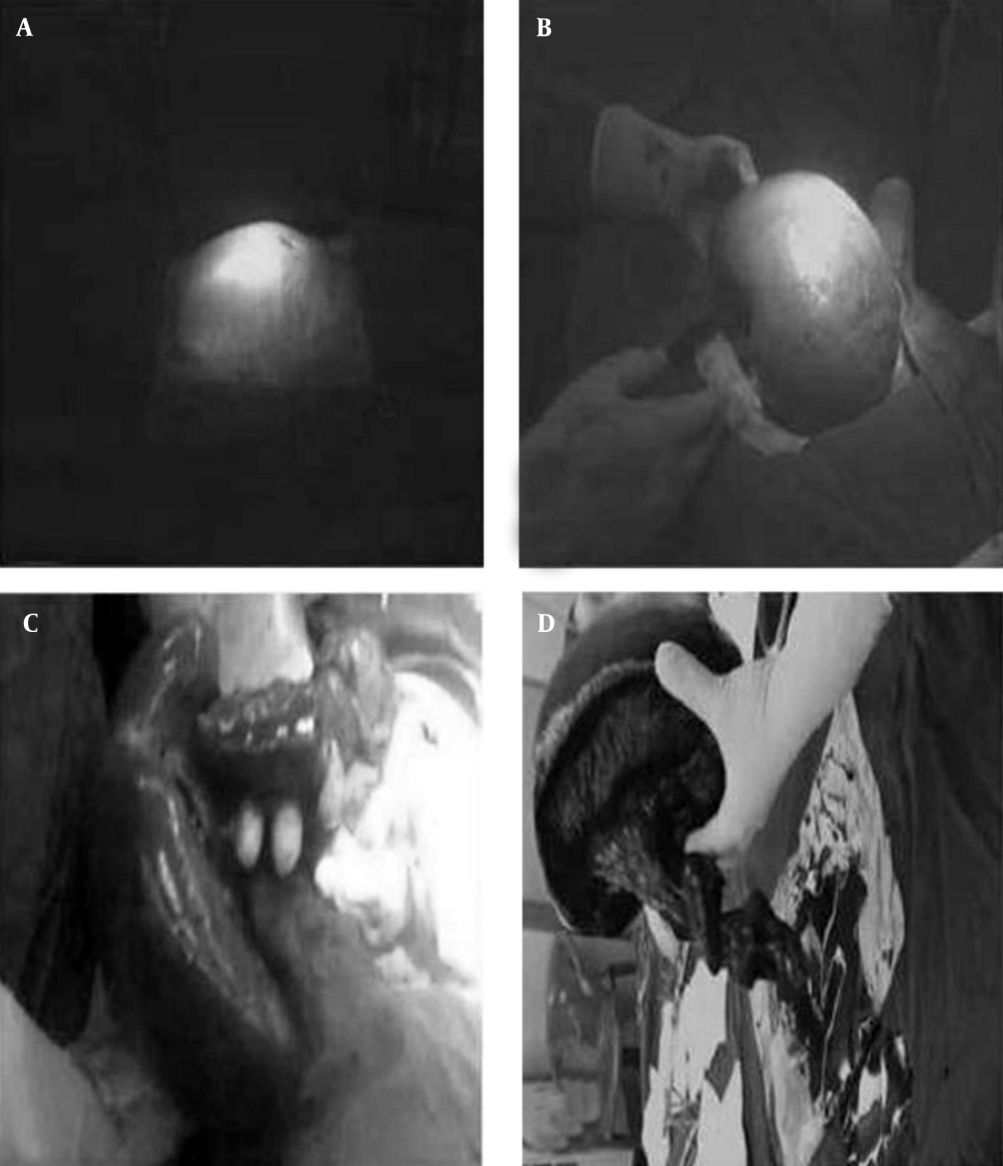
Photograph Taken During Splenectomy A) Distension of abdomen in hypogastric region; B) An enlarged spleen; C) Varicose dilatations in the veins; D) Long splenic pedicle.

A 29-year-old man with acute lower abdominal pain and vomiting was admitted to the emergency department of Shariati hospital in Isfahan province. The physical examination revealed that there is a large tender and mobile mass in hpogastric region. An abdominal distension was noticed too (Figure 1A). 

He was afebrile (36.9°C) with normal blood pressure (123/82 mm Hg) but with tachycardic (112 beats per minute). White blood cell count, hemoglobin level, platelets count and urinary analysis were normal. A plain abdominal radiographic examination showed marked colonic gaseous distension present in the left upper quadrant.

Sonographic examination showed a homogeneous 21 × 15 × 8 cm mass in the lower part of the abdomen and pelvis with echogenicity that was consistent with normal spleen tissue while the normal splenic bed in the left upper abdomen was empty. The left kidney was atrophic measuring 85 mm in length which is showing increased parenchymal echogenisity. Right kidney was also enlarged to 129 mm in length. The lower poles of both kidneys were deviated medially suggesting a horseshoe kidney. No sign of hydronephrosis or stone was detected in the kidneys. Laparotomy examination confirmed the clinical and ultrasound findings. The enlarged spleen mass in the lower part of the abdomen and pelvis had a 720 degree torsion around a 25-cm long splenic pedicle with a horseshoe kidney. The spleen had no holding ligaments. Although a detorsion of the spleen was attempted, there was no success since the pedicle had become fibrotic. There were varicose dilatations in the veins surrounding the pedicle of the spleen. The splenic artery was double tied and excised.Then the splenectomy completed by double tying and excising the vein as well. The excised spleen had 843 g weight. (Figure 1B, C, D). The patient had no additional problems during the follow-up period and was discharged three days later. 

## 3. Discussion

This was the first report on a wandering spleen associated with horseshoe kidney in an adult man. Embryologically, the spleen originates from the mesenchymal remnant of the dorsal mesogastrium at the left upper quadrant of the abdomen. During this process, the spleen establishes its peritoneal connections with the left kidney and stomach by the splenorenal and gastrosplenic ligaments (8). When these ligaments are congenitally absent or abnormally elongated, the spleen becomes wanderlust. This condition has been described as wandering spleen, floating spleen, or splenic ptosis (9, 10).

Van Horne described a case of wandering spleen for the first time in 1667 while performing an autopsy (2). The cause of this phenomena is not precisely known, but the most common cause appears to be a failure of fusion of the dorsal mesogastrium during the fifth and sixth week of development resulting in an unusually long splenic pedicle (10). Wandering spleen has also been seen in some disorders where there is failure of foregut rotation and fusion of the dorsal mesogastrium, such as prune-belly syndrome (11, 12). Other causes of wandering spleen include postpartum laxity, splenomegaly, previous abdominal trauma, poor abdominal tone and surgery (13-15). The wandering spleen is mostly found in children and in women of reproductive age (16, 17). The wandering spleen in our report is of an adult man and might be due to an embryonic anomaly, splenomegaly or post traumatic condition.

Clinically, patients may present with normal complaints, such as occasional nausea, vomiting or mild cramp-like pain due to splenic congestion or intermittent torsion and spontaneous detorsion (6, 7). Because of these nonspecific symptoms, preoperative diagnosis of wandering spleen is rarely suggested based on clinical findings alone. Therefore, imaging methods such as plain radiography, sonography, Doppler sonography, computed tomography, and MRI may play a major role in establishing the diagnosis (18-23).

Surgical treatments include splenectomy and splenopexy. For many years, the preferred treatment was to remove the spleen surgically, initially by conventional laparotomy and more recently by laparoscopy (24). The first successful splenectomy for a wandering spleen was in 1878 by Martin and marked the beginning of surgical treatment for this condition (25). Rydygier described the first successful splenopexies using various techniques and from 1890 to 1920, most authors advocated splenopexy for the treatment of this condition (26-29). Splenectomy is done if there is functional asplenia due to torsion, splenic infarction, splenic vessel thrombosis or any suspicion of malignancy. Conversely, splenopexy is preferred when a viable wandering spleen is found at laparotomy. Splenic preservation is especially recommended in extremely young patients who are at particular risk for postsplenectomy sepsis (30). This can be performed by forming a retroperitoneal pouch in which the spleen is placed, or by inserting a Prolene or Vicryl bag that is fashioned around it and secured in the left upper quadrant (31, 32).

The wandering spleen usually is located in an anterior abdominal position and is more exposed to an abdominal impact in accidents. On the other hand, it should not be denied that misplaced spleen sometimes can be the possible reason behind wrong diagnosis in cases that experience traumatic injuries. Wondering spleen is also at risk of iatrogenic traumatic injuries during surgical or endoscopic manipulation of the colon, stomach, pancreas and other abdominal organs in such cases. An increased awareness of this condition may help in reducing the incidence of mortality in emergency departments.
